# Gossypol Induces Apoptosis of Human Pancreatic Cancer Cells via CHOP/Endoplasmic Reticulum Stress Signaling Pathway

**DOI:** 10.4014/jmb.2110.10019

**Published:** 2022-02-23

**Authors:** Soon Lee, Eunmi Hong, Eunbi Jo, Z-Hun Kim, Kyung June Yim, Sung Hwan Woo, Yong-Soo Choi, Hyun-Jin Jang

**Affiliations:** 1 Division of Analytical Science, Korea Basic Science Institute, Daejeon 34133, Republic of Korea; 2 Department of Life Science and Research Institute for Natural Sciences, College of Natural Sciences, Hanyang University, Seoul 04763, Republic of Korea; 3 Microbial Research Department, Nakdonggang National Institute of Biological Resources, Sangju 37242, Republic of Korea; 4 Department of Biological Engineering, Inha University, Incheon 22212, Republic of Korea; 5 Department of Biotechnology, CHA University, Seongnam 13488, Republic of Korea; 6 Laboratory of Chemical Biology and Genomics, Korea Research Institute of Bioscience and Biotechnology, Daejeon 34141, Republic of Korea

**Keywords:** Gossypol, pancreatic cancer, apoptosis, CCAAT/enhancer-binding protein homologous protein, endoplasmic reticulum stress

## Abstract

Gossypol, a natural phenolic aldehyde present in cotton plants, was originally used as a means of contraception, but is currently being studied for its anti-proliferative and anti-metastatic effects on various cancers. However, the intracellular mechanism of action regarding the effects of gossypol on pancreatic cancer cells remains unclear. Here, we investigated the anti-cancer effects of gossypol on human pancreatic cancer cells (BxPC-3 and MIA PaCa-2). Cell counting kit-8 assays, annexin V/propidium iodide staining assays, and transmission electron microscopy showed that gossypol induced apoptotic cell death and apoptotic body formation in both cell lines. RNA sequencing analysis also showed that gossypol increased the mRNA levels of CCAAT/enhancer-binding protein homologous protein (CHOP) and activating transcription factor 3 (ATF3) in pancreatic cancer cell lines. In addition, gossypol facilitated the cleavage of caspase-3 via protein kinase RNA-like ER kinase (PERK), CHOP, and Bax/Bcl-2 upregulation in both cells, whereas the upregulation of ATF was limited to BxPC-3 cells. Finally, a three-dimensional culture experiment confirmed the successful suppression of cancer cell spheroids via gossypol treatment. Taken together, our data suggest that gossypol may trigger apoptosis in pancreatic cancer cells via the PERK-CHOP signaling pathway. These findings propose a promising therapeutic approach to pancreatic cancer treatment using gossypol.

## Introduction

Pancreatic cancer has one of the highest mortality rates among all other diseases [1-4]. Carioli *et al*. predicted over 90,000 deaths due to pancreatic cancer in the United States and Europe in 2021 [5-7]. Patients with pancreatic cancer show metastasis in 85% of cases at the time of diagnosis [8-11]. Moreover, despite improvements in diagnosis and therapy, pancreatic cancer is the only cancer that maintains a 5-year survival rate of 9% [12-15]. Thus, the discovery of effective therapeutic agents and treatment strategies is crucial.

Gemcitabine and cisplatin are two traditional drugs that have been used as a standard treatment for pancreatic cancer despite having severe side effects such as vomiting, nausea, leukopenia, and skin rash [16-18]. Owing to these side effects, as well as the development of tumor resistance to chemotherapeutic agents, there is an urgent need to identify natural compounds capable of replacing the existing drugs [18, 19]. Gossypol, a yellow polyphenolic compound present in cottonseed [20, 21], was originally utilized in China for contraception [20-23]. However, studies have demonstrated that gossypol exerts anti-cancer activity via DNA damage and apoptosis while also reducing cancer cell invasion, motility, and angiogenesis [20, 22, 24, 25]. Although the anti-cancer effects of gossypol have been evaluated in various cancers such as ovarian cancer, leukemia, and lung cancer, the intracellular mechanisms of the anti-cancer effects of gossypol in pancreatic cancer cells remain unclear.

Since pancreatic cells contain an enlarged endoplasmic reticulum (ER) due to secretion of digestion enzymes and insulin [26-28], they are likely to be highly sensitive to ER stress-induced apoptosis. In addition to secretion-related functions, the ER plays a crucial role in protein regulation, including synthesis, folding, and modification [29-31]. The imbalance of intracellular Ca^2+^ and the accumulation of unfolded and misfolded proteins can lead to ER stress [32-34], which can then be detected by ER transmembrane receptors such as protein kinase RNA-like ER kinase (PERK), activating transcription factor 6 (ATF6), and inositol-requiring enzyme 1 (IRE1) [35-38]. CCAAT/enhancer-binding protein homologous protein (CHOP), a downstream protein of PERK, was first identified as a mediator that induces apoptosis in response to ER stress [39-42]. Therefore, the induction of apoptosis in pancreatic cancer cells via ER stress represents an effective approach for treating pancreatic cancer compared to the treatment of other cancers.

In this study, we investigated whether gossypol activates the apoptosis-mediated ER stress pathway in two pancreatic cancer cell lines, BxPC-3 cells containing wild-type Kirsten Ras proto-oncogene (KRAS), and MIA PaCa-2 cells containing mutant KRAS [43-45]. We evaluated the apoptosis-inducing effect of gossypol based on cytotoxicity, observation of morphological changes and intracellular structures, and annexin V/propidium iodide (PI) staining. Additionally, we determined the effect of gossypol on normal human mesenchymal stem cells (hMSCs). The mechanism of action of gossypol’s effects related to ER stress-induced apoptosis was assessed by analyzing the mRNA as well as the protein expression levels of CHOP, ATF4, and PERK. Finally, we confirmed the anti-cancer effects of gossypol in three-dimensional (3D) pancreatic cancer cell spheroid cultures.

## Materials and Methods

### Reagents and Chemicals

Gossypol was purchased from Sigma-Aldrich (USA). Antibiotic-antimycotic solution (100×), 0.25% trypsin-ethylenediaminetetraacetic acid (EDTA) solution, and phosphate-buffered saline (PBS) were procured from Gibco (USA). Roswell Park Memorial Institute (RPMI) medium, Dulbecco’s Modified Eagle’s Medium (DMEM), and fetal bovine serum (FBS) were purchased from PAN-Biotech (Germany). Muse Annexin V & Dead Cell reagent was obtained from Millipore (USA). Whole cell lysis buffer was procured from iNtRON Biotechnology (Korea).

### Cell Lines and Cell Viability Assay

Human pancreatic cancer cell lines BxPC-3 and MIA PaCa-2 were purchased from the American Type Culture Collection (ATCC) (USA). They were cultured in RPMI medium and DMEM supplemented with 10% (v/v) FBS and 1% (w/v) antibiotic-antimycotic at 37°C with 5% (v/v) CO_2_. Human mesenchymal stem cells (hMSCs) were purchased from Lonza (USA) and cultured in StemMACS MSC expansion media (Miltenyi Biotec, Germany). Cells (5 × 10^3^ cells/well) were seeded in 96-well plates and incubated for 24 h before gossypol treatment for 24, 48, and 72 h. At the end of each time point, 10 μl of Cell Counting Kit-8 solution (DojinDo, Japan) was added to each well, and the plates were incubated for 90 min at 37°C. Cell viability was quantified by measuring the absorbance of the solution at 450 nm using a microplate reader (Sunrise, Tecan, Switzerland). The assay was performed in triplicate. The half-maximal inhibitory concentration was determined after evaluating the cytotoxicity of gossypol at 24, 48, and 72 h.

### Detection of Apoptosis Using Annexin V/PI Staining 

Flow cytometry analysis was performed using the Annexin V-Fluorescein Isothiocyanate (FITC) Apoptosis Detection Kit (Sigma-Aldrich). The cells were treated with various concentrations of gossypol for 24 and 48 h, detached using trypsin, and washed twice with PBS. The cell suspension in PBS was centrifuged at 200 ×*g* for 5 min, and the supernatant was carefully removed via pipetting. The cell pellet was suspended in 500 μl annexin V binding buffer, to which 0.1 μg/ml annexin V-FITC conjugate and 2 μg/ml PI were added, and the cells were then incubated for 10 min at room temperature in the dark. The fluorescence of the samples was detected using a Guava Flow Cytometry System (Millipore) at an excitation wavelength of 488 nm using a 530/30 nm band-pass filter to detect annexin V and a 670 nm high-pass filter to detect PI.

### RNA Sequencing

RNA sequencing was performed as described in our previous study [13]. RNA was extracted using TRIzol reagent (Invitrogen, USA) and analyzed with an Agilent 2100 bioanalyzer (Agilent Technologies, The Netherlands). Libraries were prepared from total RNA using the NEBNext Ultra II Directional RNA-Seq Kit (New England Biolabs, UK). The isolated mRNA was used for cDNA synthesis and shearing, following the manufacturer’s instructions. Indexing was performed using the Illumina indexes 1–12, and polymerase chain reaction (PCR) was performed in the enrichment step. A library quantification kit and a StepOne Real-Time PCR System (Life Technologies, USA) were used for quantification. High-throughput sequencing was performed as paired-end 100 sequencing using the HiSeq X10 system (Illumina, USA). For data analysis of RNA sequencing, quality control of raw sequencing data was performed using FastQC (https://www.bioinformatics.babraham.ac.uk/projects/fastqc/). Adapter and low-quality reads (<Q20) were removed using FASTX_Trimmer (http://hannonlab.cshl.edu/fastx_toolkit/) and BBMap (https://sourceforge.net/projects/bbmap). The trimmed reads were then mapped to the reference genome using TopHat [46]. Gene expression levels were estimated using Fragments Per Kilobase Million (FPKM) mapped reads from Cufflinks [47]. The FPKM values were normalized via the quantile normalization method using EdgeR within R (https://www.r-project.org/). Data mining and graphic visualization were performed using ExDEGA (E-Biogen, Korea).

### Gene Ontology-Based Network Analysis

The biological functions of the regulated genes were determined via an interaction network prepared using the STRING database (http://string-db.org/). The biological functions of differentially expressed genes and proteins were evaluated according to ontology-related interaction networks, including apoptosis signaling. Network generation was optimized based on the obtained expression profiles and was set to produce highly connected networks.

### Immunoblotting

Total cell lysates were prepared by lysing cells in 1 ml of Tris-HCl (20 mM) containing a protease inhibitor cocktail (Roche, Switzerland), which was then placed on ice for 30 min, and centrifuged for 10 min (10,000 ×*g*, 4°C). The protein content in the cell lysates was quantified using the bicinchoninic acid assay [48]. Denatured proteins (30 μg) were separated using 12% sodium dodecyl sulphate-polyacrylamide gel electrophoresis and transferred onto a 0.2 μm polyvinylidene fluoride membrane that was submerged in transfer buffer for 3 h. The membrane was blocked for 1 h using 5% (w/v) skimmed milk in Tris-buffered saline containing Tween-20 (TBST) and incubated with the following antibodies: PERK, phosphorylated-PERK, ATF4, CHOP, caspase-3, cleaved caspase-3, BCL-2, and BAX (Cell Signaling Technology, USA), and glyceraldehyde-3-phosphate dehydrogenase (GAPDH) (Santa Cruz Biotechnology, USA). After three washes (10 min each) with TBST, the membrane was incubated with horseradish peroxidase-conjugated goat anti-mouse or rabbit anti-goat IgG (Santa Cruz Biotechnology) in TBST containing 5% (w/v) skimmed milk at room temperature for 1 h. The membrane was rinsed thrice (10 min each) with 0.1% (v/v) TBST. An enhanced chemiluminescence system (Thermo Fisher Scientific, USA) was used to visualize the bands using the ChemiDoc MP System (Bio-Rad, USA). Densitometry of the bands was performed using ImageJ software (National Institutes of Health, USA). Protein levels were quantitatively analyzed and normalized against GAPDH.

### 3D Spheroid Culture

Cells were seeded onto 96-well, ultra-low attachment microplates (500 cells/well) and centrifuged at 200 ×*g* for 5 min. Spheroids were allowed to form for 4 days after seeding prior to gossypol treatment at various concentrations. On day 3 of treatment, the medium was replaced with fresh gossypol-containing medium. Morphological changes in the spheroids were observed for a week, and the volume of the spheroids was evaluated using NIS-Elements imaging software (Nikon, Japan).

### Statistical Analyses

GraphPad Prism (GraphPad Software, USA) was used to perform all statistical analyses. All measurements were performed in triplicate, and all values were expressed as the mean ± SEM. The results were processed via analysis of variance using the Tukey’s test to assess the statistical significance of differences between groups. A value of *p* < 0.05 was considered significant.

## Results

### Gossypol Treatment Sensitively Induced Cell Death in Pancreatic Cancer Cells Than in Normal Cells

**Fig. 1:**
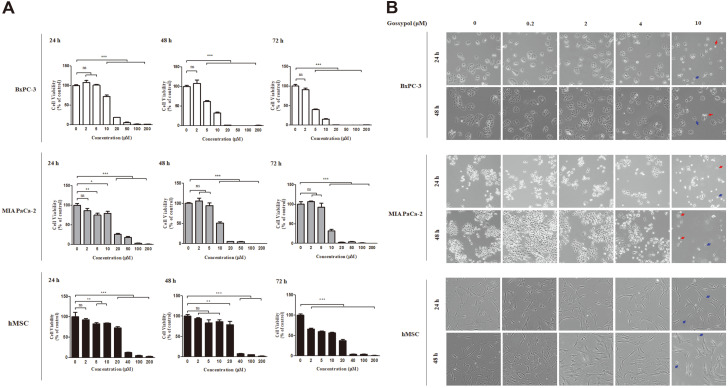
Gossypol treatment sensitively induced cytotoxicity of BxPC-3 and MIA PaCa-2 pancreatic cancer cells contrary to human mesenchymal stem cells (hMSCs). Cytotoxic effect of pancreatic cancer cells via gossypol treatment. (**A**) BxPC-3, MIA PaCa-2 and hMSCs were exposed to 0, 2, 5, 10, 20, 100, and 200 μM gossypol for 24, 48, and 72 h. (**B**) Morphological changes in BxPC-3, MIA PaCa-2 and hMSCs after treatment with indicated concentrations of gossypol for 24 h and 48 h observed via microscopy (100× magnification). Blue arrow indicates shrunken cells and red arrow shows floating cells. Data represent the mean ± SEM of three independent experiments. *p < 0.05, **p < 0.01, and ***p < 0.001 versus non-treated cells.

To determine how gossypol changes the cytotoxicity of human pancreatic cancer cells, BxPC-3, MIA PaCa-2 cells and hMSCs were treated with 0, 2, 5, 10, 20, 50, 100, and 200 μM gossypol for 24, 48, and 72 h. Gossypol treatment reduced viability in a dose- and time-dependent manner in pancreatic cancer cell lines; cytotoxicity was induced by approximately 20% via treatment with 10 μM gossypol for 24 h (Fig. 1A). Exposure to 5 μM of gossypol for 48 h induced toxicity in BxPC-3 cells by 50%, whereas no death inhibition was observed in MIA PaCa-2 cells. However, 20 μM gossypol treatment for 48 h promoted only 20% death in hMSC, compared with 99% in BxPC-3 and 92% in MIA PaCa-2. The half-maximal inhibitory concentration (IC50) of gossypol during 1 day on BxPC-3, MIA PACa-2 and hMSC is 14, 15, and 28 μM. The IC50 of each cell line for 2 days is 6, 10, and 30 μM, and 4, 8, and 20 μM for 3 days.

The morphological changes in these cells were observed using a light microscope after exposure to different concentrations of gossypol (0, 0.2, 2, 4, and 10 μM). Treatment with 10 μM gossypol for 24 and 48 h induced drastic morphological changes in both pancreatic cancer cell lines (Fig. 1B). While the hMSCs revealed shrunken morphology, the cancer cells exhibited a reduced cell volume and appeared buoyant. These surface structural changes induced via gossypol treatment preceded apoptosis. Thus, these results confirmed that gossypol may effectively inhibit the proliferation of pancreatic cancer cells.

### Gossypol Treatment Induced Damaged Intracellular Structures.

**Fig. 2:**
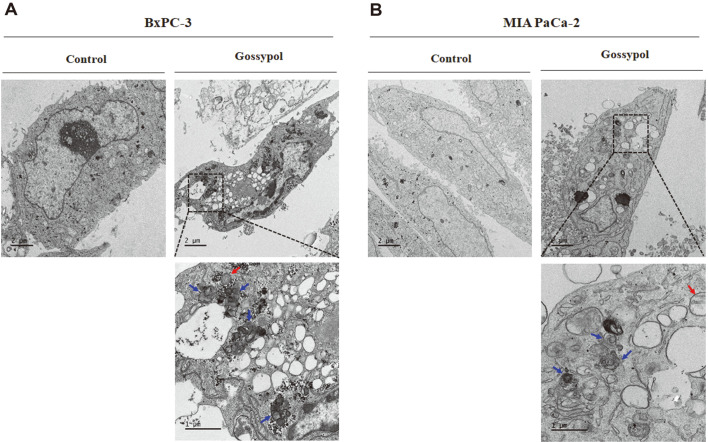
Observation of intracellular structural changes and apoptotic bodies in gossypol-treated pancreatic cancer cells via TEM. (**A**) BxPC-3 and (**B**) MIA PaCa-2 cells were analyzed via TEM after treatment with vehicle- or gossypol (4 μM) for 24 h. Representative images are shown. Blue arrow indicates autophagosomes and red arrow means damaged mitochondria. TEM, transmission electron microscopy.

We performed transmission electron microscopy (TEM) to visualize the intracellular changes in pancreatic cancer cells in response to gossypol treatment. After 24 h treatment with 4 μM gossypol, cells exposed to gossypol showed damaged mitochondria and autophagosomes containing dense organelles (Fig. 2). These cells also presented abnormal spherical vacuoles containing fragmented and segregated chromatin clumps. Conversely, untreated BxPC-3 and MIA PaCa-2 cells presented intact plasma membranes and did not contain vesicle-like structures.

### Gossypol Treatment Promoted Apoptotic Cell Death in Pancreatic Cancer Cells.

**Fig. 3:**
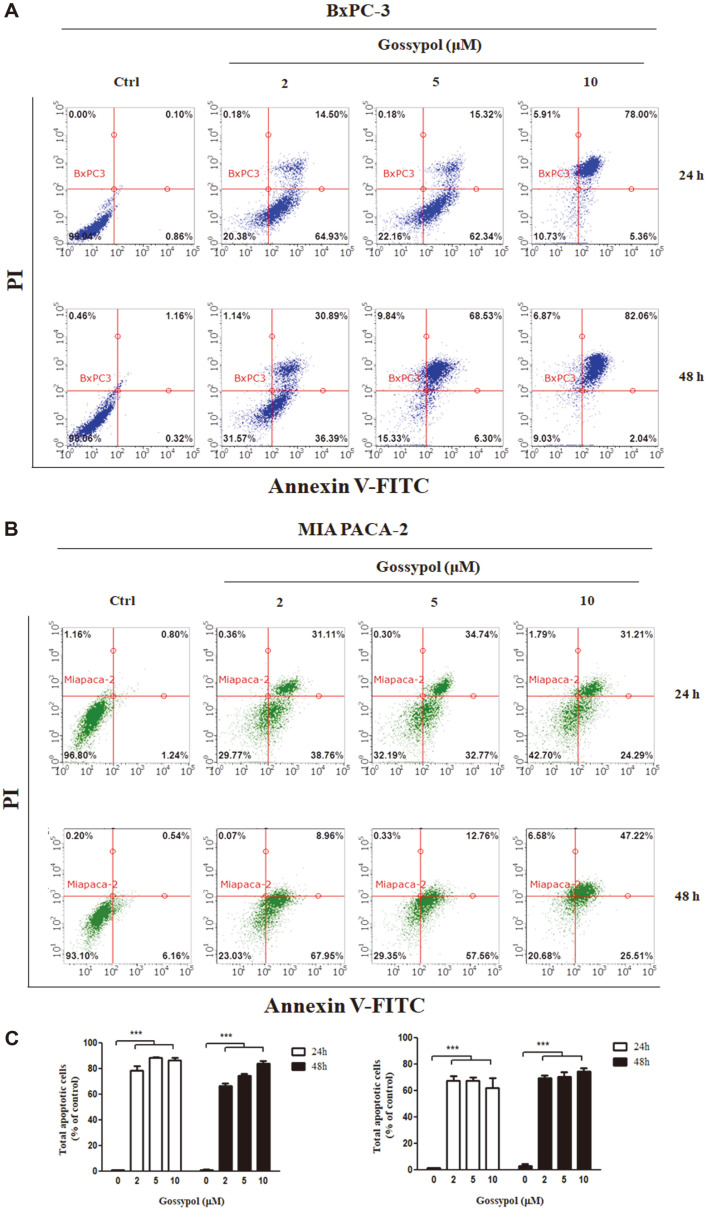
Analysis of apoptosis in pancreatic cancer cells after treatment with gossypol at different concentrations. (**A**) BxPC-3 and (**B**) MIA PaCa-2 cells were stained with fluorescent dyes and analyzed using a Guava Flow Cytometry System using the Annexin V-Fluorescein Isothiocyanate Apoptosis Detection Kit. Values are expressed as a percentage of early and late apoptotic cells. (**C**) Data represent the mean ± SEM of three independent experiments.

We hypothesized that the anti-proliferative effect and morphological alteration induced by gossypol are mediated via apoptotic cell death in BxPC-3 and MIA PaCa-2 cells. Therefore, to investigate whether gossypol treatment induced apoptotic cell death in pancreatic cancer cells, we performed annexin V/PI staining and flow cytometric analysis for cells treated with gossypol (0, 2, 5, and 10 μM) for 24 h and 48 h (Fig. 3). The relative fractions of cells in the early stage of apoptosis (annexin V-stained, non-disrupted cells) and the late stage of apoptosis (disrupted or lysed cells) were determined. Treatment with gossypol (2 μM) reduced the proportion of viable BxPC-3 cells (99% to 20%) and MIA PaCa-2 cells (96.8% to 29.7%) in 24 h. In response to 48 h of treatment (10 μM), the proportion of cells in the apoptotic stage markedly increased from 1.4% to 84.0% in BxPC-3 cells and from 6.6% to 72.7% in MIA PaCa-2 cells. In addition, the number of viable cells significantly decreased (98% to 9%in BxPC-3 cells and 93% to 20% in MIA PaCa-2 cells). These results indicated that gossypol treatment might have triggered apoptotic cell death in BxPC-3 and MIA PaCa-2 cells.

### Gossypol Treatment Modulated the Gene Expression in Human Pancreatic Cancer Cells

**Fig. 4:**
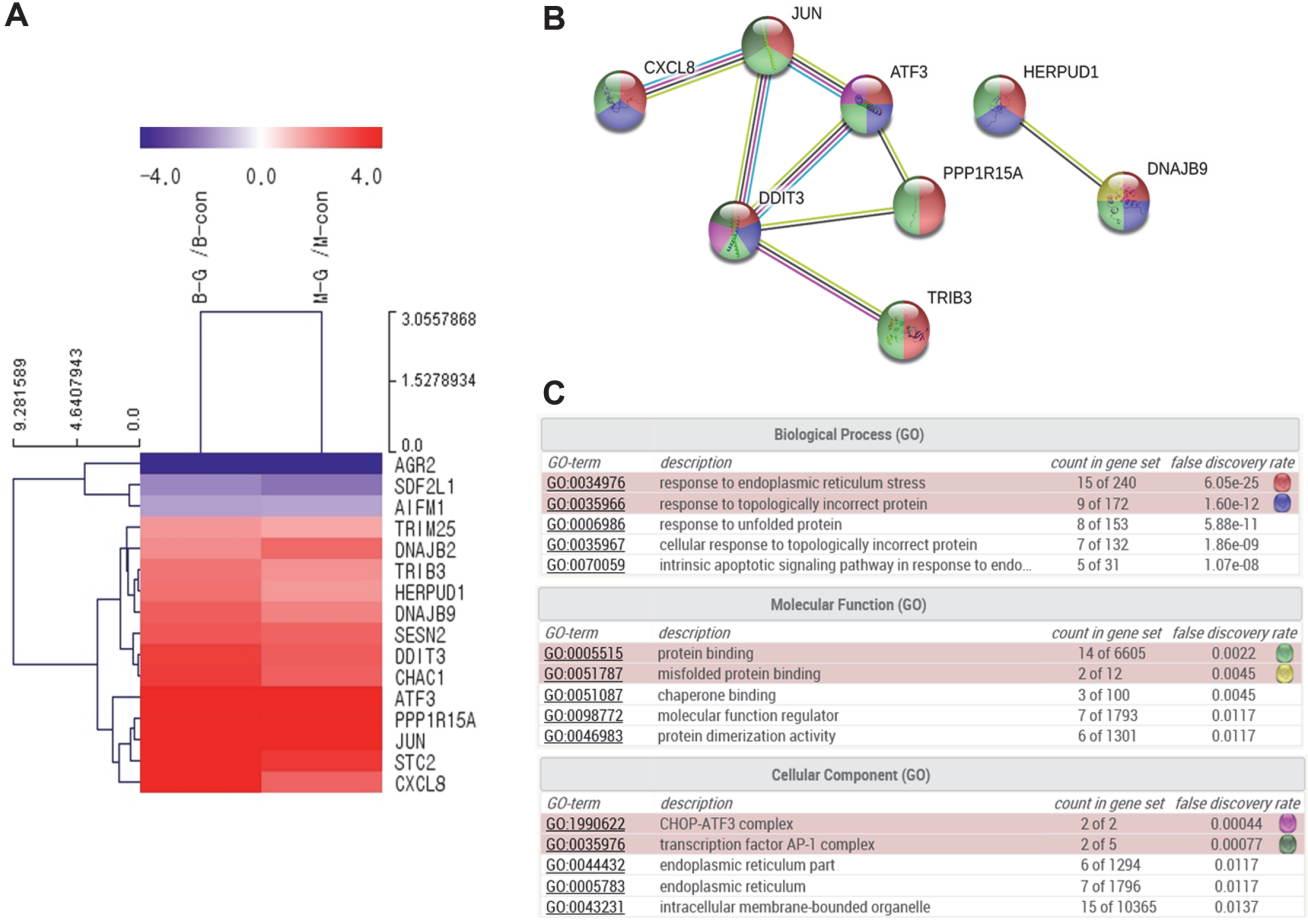
RNA sequencing analysis of the gene expression changes and signaling network in gossypol-treated pancreatic cancer cells. Hierarchical gene clustering was performed using the TM4 Microarray Software Suite (MeV) for gossypol-treated BxPC-3 and MIA PaCa-2 pancreatic cancer cells. Red and blue colors represent genes that were up- and downregulated compared to control group, respectively, by more than 2.5-fold. The ratios of gene profiles are presented as a (**A**) heatmap and gene expression pattern. (**B**) Combined screenshots from the STRING website showing results obtained upon analyzing a set of 8 proteins suspected to be involved in the apoptotic process (GO analysis). The insets show the accessory information available for a single protein, a reported enrichment of functional connections among the set of proteins, and statistical enrichments detected in functional subsystems. (**C**) GO analysis of protein–protein interactions. Enriched functions were selected and the corresponding protein nodes in the network were automatically highlighted. GO, gene ontology.

Since gossypol treatment increased apoptotic cell death in pancreatic cancer cells, we performed RNA sequencing for BxPC-3 and MIA PaCa-2 cells treated with 4 μM gossypol for 24 h to identify the intracellular pathway associated with gossypol-mediated effects. Of the 24,424 unique genes evaluated, the expression of 595 genes was altered in gossypol-treated cells, among which 254 were upregulated and 341 were downregulated. We used the Excel-based differentially expressed gene analysis (ExDEGA) program [49] to combine the biological features of genes and categorize genes that play a crucial role in apoptosis. The ER stress-related genes that were up- and downregulated by 2.5-fold in gossypol-treated BxPC-3 and MIA PaCa-2 cells were analyzed using the Multiple Experiment Viewer (MeV) tool and hierarchical cluster analysis (Fig. 4A). We found that gossypol treatment modulated the expression of 16 genes, among which 3 genes were downregulated and 13 genes were upregulated. Gossypol treatment triggered apoptosis by upregulating DNA damage-inducible transcript 3 (*DDIT3*, also known as CHOP) (Fig. 4B). Additionally, anterior gradient protein 2 (*AGR2*) was downregulated in gossypol-treated BxPC-3 and MIA PaCa-2 cells.

Based on these results, we analyzed the protein-protein interactions and gene ontology using the STRING database (Fig. 4C). We analyzed the network of eight genes including *DDIT3* and *ATF3*. Gossypol treatment modulated the apoptotic process by regulating ER stress (GO: 0034976; false discovery rate P = 6.05e−25: CHOP, ATF3, protein phosphatase 1 regulatory subunit 15A (PPP1R15A), chemokine ligand 8 (or interleukin 8), JUN, DnaJ homolog subfamily B member 9, and homocysteine-responsive ER-resident ubiquitin-like domain member 1 protein).

### Gossypol Treatment Increased Caspase-3 Cleavage by Upregulating PERK and CHOP

**Fig. 5:**
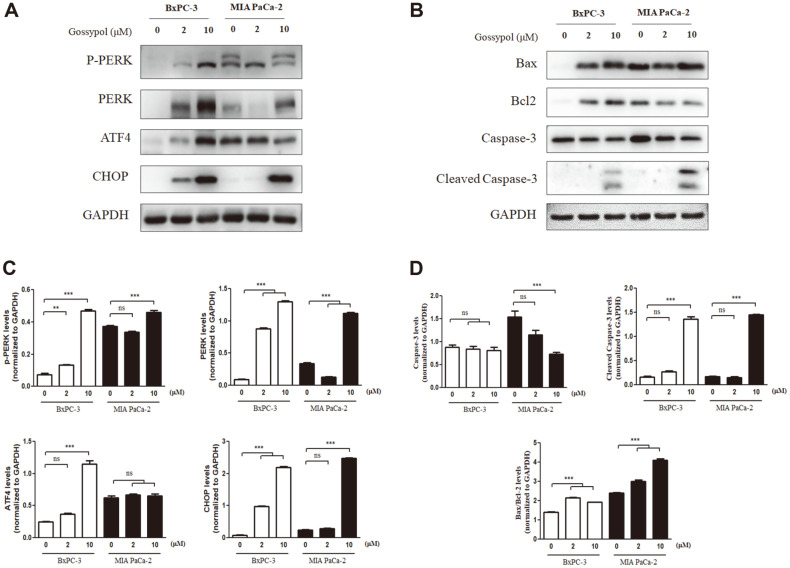
Gossypol treatment induces mitochondrial apoptosis with endoplasmic reticulum stress pathway via PERK and CHOP signaling in pancreatic cancer cells. Treatment of gossypol (0, 2, and 10 μM) for 24 h increased (**A**) p-PERK, PERK, CHOP, (**C**) Bax/Bcl-2, and cleaved caspase-3 protein expression in BxPC-3 and MIA PaCa-2 cells. (**B** and **D**) The band densities were quantified and normalized to GAPDH. Data represent the mean ± SEM of three independent experiments. *p < 0.05, **p < 0.01, and ***p < 0.001 versus non-treated cells. PERK, protein kinase RNA-like ER kinase; CHOP, CCAAT/enhancer-binding protein homologous protein; p-PERK, phosphorylated PERK; GAPDH, glyceraldehyde-3- phosphate dehydrogenase.

Western blotting was performed to evaluate changes in the expression of ER stress-related proteins in gossypol-treated BxPC-3 and MIA PaCa-2 cells. Treatment with 0, 2, and 10 μM gossypol for 24 h resulted in an increase in PERK and CHOP protein levels in both cell lines. Particularly in BxPC-3 cells, a drastic increase of over 10-fold was observed after 10 μM of gossypol treatment, and ATF4 upregulation was also evident (Figs. 5A and 5C).

We then assessed the expression levels of apoptosis-related proteins such as caspase-3, B-cell lymphoma 2 (Bcl-2), and Bcl-2-associated X (Bax). In both cell lines, gossypol treatment increased the levels of cleaved caspase-3 and the Bax/Bcl-2 ratio by approximately 8- and 1.5-fold, respectively (Figs. 5B and 5D). Moreover, gossypol did not induce cleaved caspase-3 in BxPC-3 and MIA PaCa-2 cells, which are blocked by PERK or CHOP expression (Fig. S1). Together, our results indicate that gossypol treatment may trigger pancreatic cancer cell death via the ER stress-related PERK-CHOP signaling pathway.

### Gossypol Treatment Inhibited the Growth of BxPC-3 and MIA PaCa-2 Spheroids in 3D Culture

**Fig. 6:**
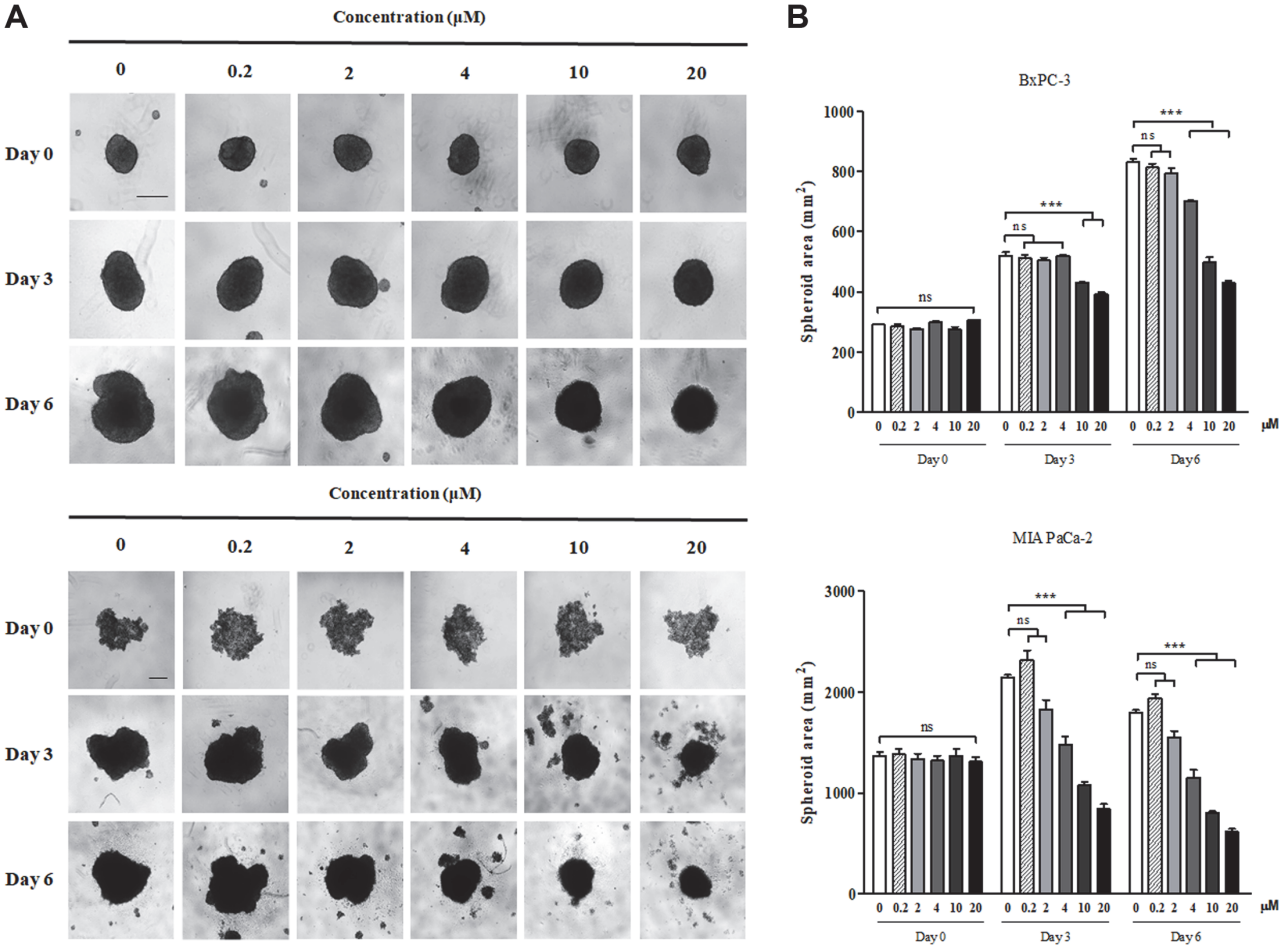
Growth inhibitory effect of gossypol on three-dimensional cultured BxPC-3 and MIA PaCa-2 cells. Cells were cultured for 4 d post-seeding to allow spheroid formation before drug treatment. (**A**) Representative microscopic images of spheroids on day 0, 3, and 6 of treatment. Scale bar represents 500 μm. (**B**) Area of spheroids on day 0, 3, and 6 of treatment. Data represent the mean ± SEM of three independent experiments. ***p < 0.0001 vs. vehicle cells.

Since cancer cells grow in 3D multilayers in a body instead of two-dimensional (2D) monolayers, 2D cultured cells differ from actual tumors [50, 51]. To account for this phenomenon, we sought to determine whether gossypol treatment reduced the 3D spheroid volume of BxPC-3 and MIA PaCa-2 cells. Four days post-seeding, spheroids were visualized and treated with different doses of gossypol (day 3) (Fig. 6). The BxPC-3 spheroids continued to grow larger in size during the treatment period; however, the growth rate was slower when treated with gossypol. Growth inhibition was significant in the 10 and 20 μM treatments on day 3, and was significant in the 4, 10, and 20 μM treatments on day 6. The area of MIA PaCa-2 spheroids decreased when treated with concentrations of 4, 10, and 20 μM of gossypol, and there were significant differences compared to the area of untreated spheroids (Fig. 6B).

## Discussion

Cancer cells constantly experience intra- and extra-cellular stresses such as hypoxia, nutrient deprivation, and compression from the surrounding microenvironment [52]. As cancer cells proliferate incessantly, they are under a constant stress to proliferate, synthesize proteins, and maintain homeostasis [53, 54]. To withstand such stress conditions and sustain growth, these cells activate the ER stress mechanism, which triggers the unfolded protein response by inducing IRE1, PERK, and ATF6 expression [36, 54, 55]. Therefore, modulation of ER stress signaling to activate the apoptosis pathway is a key consideration and promising therapeutic approach for the effective treatment of cancer. In this regard, we focused on the ER stress-mediated apoptosis-inducing effect of gossypol on the human pancreatic cancer cell lines BxPC-3 and MIA PaCa-2, which has not been studied extensively.

Mohammad *et al*. investigated the anticancer effect of gossypol in pancreatic cancer and only validated mitochondrial apoptosis in BxPC-3 cells containing wild-type KRAS induced by gossypol, without performing a mechanistic study [56]. This is not sufficient to evaluate gossypol as a therapeutic reagent for pancreatic cancers, since approximately 90% of developing pancreatic cancer cells contain mutant KRAS [57]. Hence, we examined the intracellular mechanism of action of gossypol in both BxPC-3 and MIA PaCa-2 cells, which express contrasting KRAS genotypes, and focused on the ER stress pathway [28, 43, 44]. We found that gossypol treatment suppressed proliferation and induced apoptotic morphological changes, including shrinkage, detachment, and damaged mitochondria [58, 59]. Since cells expose phosphatidylserine via a broken membrane structure during apoptosis, annexin V, which binds to phosphatidylserine, serves as a marker for apoptosis [60]. We confirmed that gossypol treatment induced annexin V-positive cell death, but not PI-positive cell death, indicating non-apoptotic cell death (Fig. 3). However, gossypol affected normal cells, hMSCs, less sensitively. Thus, our data demonstrated that gossypol may trigger apoptotic cell death in pancreatic cancer cells exhibiting wild-type and mutant KRAS with low toxicity to normal cells.

Previous studies showed that gossypol downregulates inflammation genes such as those related to tristetraprolin, interleukins, and diacylglycerol acyltransferases in colon cancer [45]. Gossypol upregulates apoptosis-promoting genes such as those involved in growth arrest and DNA damage-inducible 45 alpha (*GADD45A*), tumor necrosis factor receptor superfamily 9, and BCL2-interacting protein 3 in breast cancer cells [61]. In our study, we found that gossypol treatment markedly upregulated the expression of PPP1R15A, Tribbles-related protein 3 (TRIB3), and ATF3 in pancreatic cancer cells (Fig. 4). PPP1R15A, also known as GADD34, is a protein downstream of CHOP that alleviates ER stress by stopping protein synthesis and mediating cell recovery [34, 62-64]. TRIB3 is an ER stress-inducible protein that functions downstream of ATF4 and CHOP to facilitate apoptosis [35-37, 65-67]. Therefore, although gossypol promotes apoptosis in various cancer cells, ER stress signaling is induced specifically in pancreatic cancer cells via regulation of *PPP1R15A*, *TRIB3*, and *ATF3* gene expression. In addition, the greater increase in TRIB3 expression via gossypol treatment in BxPC-3 cells than that in MIA PaCa-2 cells may not only correlate with higher toxicity, but could also result in a better therapeutic effect against pancreatic cancer cells containing wild-type KRAS.

ATF3 is necessary for death receptor 5 (DR5) induction by ER stress in p53-deficient cancer cells [68-70]. Since BxPC-3 and MIA PaCa-2 cells possess mutated p53, it is likely that gossypol might have activated the rapid expression of DR5 and facilitated apoptotic cell death via ATF3 upregulation [44, 70, 71].

Among the proteins involved in the ER stress pathway, ATF4 is known to function as a dual effector in the life-and-death decisions of cells [72, 73]. Along with the DR5 protein, ATF4 plays an important role in mitochondrial apoptosis via glucose depletion-induced ER stress and acts as a mediator of the PERK and CHOP pathways of apoptosis [72, 74, 75]. *ATF4* mRNA levels were increased via the PI3K-AKT pathway by KRAS mutations, as a markedly higher level of basal ATF4 expression was observed in MIA PaCa-2 cells than that in BxPC-3 cells (Figs. 5A and 5C). Increased ATF4 levels activate mechanistic target of rapamycin complex 1, leading to suppression of apoptosis and promotion of cell proliferation [76-78]. This may also explain the lower cytotoxicity induced by gossypol in MIA PaCa-2 cells (Figs. 1 and 5). Additionally, autophagy increased by the KRAS mutation might have exerted a protective effect against gossypol-mediated cytotoxicity, thereby facilitating the survival of MIA PaCa-2 cells [79]. These differences in gene expression, such as in *ATF4* and *DDIT3*, may correlate with the differing cytotoxicity in BxPC-3 and MIA PaCa-2 cells. Therefore, our results suggest that the expression levels of ATF4 may affect the efficacy of drugs that target ER stress signaling in the treatment of pancreatic cancer.

Gossypol is a terpenoid aldehyde that is formed via the acetate-isoprenoid pathway [80]. According to He *et al*., the aldehyde group in gossypol promotes mitochondrial apoptosis by blocking the elimination of oxidative stress [81]. Reactive oxygen species (ROS) induce mitochondrial apoptosis via modulation of Bcl-2 family proteins such as Bax, Bcl-2, Bid, and Bak [82-84]. Additionally, PERK and CHOP, which are regulators of GADD34 and TRIB3, can regulate Bcl-2 family protein levels [85-88]. Gossypol treatment increased the levels of Bax/Bcl-2, PERK, and CHOP in the present study (Fig. 5). Therefore, the aldehyde group of gossypol may increase ROS production and induce mitochondrial apoptosis via PERK and CHOP in pancreatic cancer cells. However, since gossypol is also known for its protective function against oxidative stress through previous studies, the effect of gossypol inducing ER stress through oxidative pathway needs to be clarified [21, 89, 90].

Although in vitro cancer cells are not identical to cells in tumors, cells cultured in 3D spheroids exhibit in vivo tumor characteristics, including drug resistance, nutrient gradient, and polarity [51, 91]. The treatment of 3D cultured pancreatic cancer cells with gossypol blocked the extension of the 3D spheroidal volume, indicating that gossypol may also hinder the growth of pancreatic tumors in the body, similar to 3D pancreatic cancer cells. Moreover, gossypol only slightly reduced cell viability in normal cells, hMSCs, by comparison with pancreatic cancer cells. This vulnerability gap between normal cells and cancer cells may cause a difference in the viability against gossypol. In previous studies, hMSCs can modulate various stresses such as ER and oxidative stress, but not thermal stress [92, 93]. The exosomes derived from hMSCs decrease ER stress and protect cells from apoptosis. Additionally, hMSCs reduce ER stress themselves through the PERK-Nrf2 pathway [94]. Since enlarged ER by the secretion function of digestive enzymes, pancreatic cancer cells are specifically sensitive to ER stress [26-28]. Therefore, our data indicate the applicability of gossypol in human pancreatic cancer treatment with low side effect.

In the present study, the apoptosis-inducing effect of gossypol was investigated via cell viability assays, observation of morphological changes and intracellular structures, and annexin V/PI analysis. mRNA sequencing and western blotting analyses were performed to determine the mechanism of action of gossypol-mediated effects on pancreatic cancer cells. Finally, the anticancer effect of gossypol was confirmed by monitoring the changes in 3D cultured cancer cells to evaluate whether gossypol is effective against in vivo tumors.

Our study showed that gossypol treatment increased apoptotic death in pancreatic cancer cells by activating ER stress signaling via the upregulation of PERK and CHOP, regardless of the KRAS status. Moreover, our findings suggest that gossypol may be effective against 2D cultured cells as well as 3D spheroid pancreatic cancer cells which simulate the tumor microenvironment. Moreover, gossypol only slightly inhibited the growth of normal cells by comparison to cancer cells. Collectively, gossypol, an ER stress inducer, represents a promising candidate for use as an anticancer drug and provides an effective strategy for improving the life of patients with pancreatic cancer.

## Supplemental Materials

Supplementary data for this paper are available on-line only at http://jmb.or.kr.
