# Supplementary material for: Gossypol Induces Apoptosis of Human Pancreatic Cancer Cells via CHOP/Endoplasmic Reticulum Stress Signaling Pathway
Source: J Microbiol Biotechnol. 2022 Feb 23;32(5):645–56. doi: 10.4014/jmb.2110.10019 (PMC9628887; doi:10.4014/jmb.2110.10019)
Supplement: Supplementary file 1 [file jmb-32-5-645-supple.pdf]

J. Microbiol. Biotechnol.
https://doi.org/10.4014/jmb.2110.10019

Supporting Information

Gossypol suppresses proliferation of human pancreatic cancer cells via CHOP/endoplasmic reticulum stress signaling pathway

Soon Lee[1], Eunmi Hong[1], Eunbi Jo[2], Z-Hun Kim[3], Kyung June Yim[3], Sung Hwan Woo[4], Yong-Soo Choi[5], and Hyun-Jin Jang[6,*]

[1]Division of Analytical Science, Korea Basic Science Institute, 169-148 Gwahakro, Daejeon 34133, Republic of Korea

[2]Department of Life Science and Research Institute for Natural Sciences, College of Natural Sciences, Hanyang University, 222 Wangsimni-ro, Seoul 04763, Republic of Korea

[3]Microbial Research Department, Nakdonggang National Institute of Biological Resources, Sangju-si 37242, Gyeongsangbuk-do, Republic of Korea

[4]Department of Biological Engineering, Inha University, Incheon 22212, Korea

[5]Department of Biotechnology, CHA University, Seongnam, 13488 Republic of Korea

[6]Laboratory of Chemical Biology and Genomics, Korea Research Institute of Bioscience and Biotechnology, Daejeon 34141, Republic of Korea

***Correspondence to:** Dr. Hyun-Jin Jang (E-mail: hjjang0228@gmail.com, Laboratory of Chemical Biology and Genomics, Korea Research Institute of Bioscience and Biotechnology, 125 Gwahakro, Daejeon 34141, Republic of Korea, Tel: +82-42-860-4563)

Running title: Gossypol induces death of human pancreatic cancer

Materials and Methods

siRNA transfection

siRNAs purchased from Santa Cruz (Texas, USA) were used to target PERK and CHOP expression. Cells were seeded and cultured in serum-free media 24h prior to transfection. A mixture of Hilymax solution (DojinDo, Kumamoto, Japan) and siRNA were incubated for 15m at RT. The mixture was applied to the cells, and was exchanged with normal growth medium after 4 hours of incubation. Fresh medium was provided 24h later, and cells were treated with gossypol the following day.

Figure S1

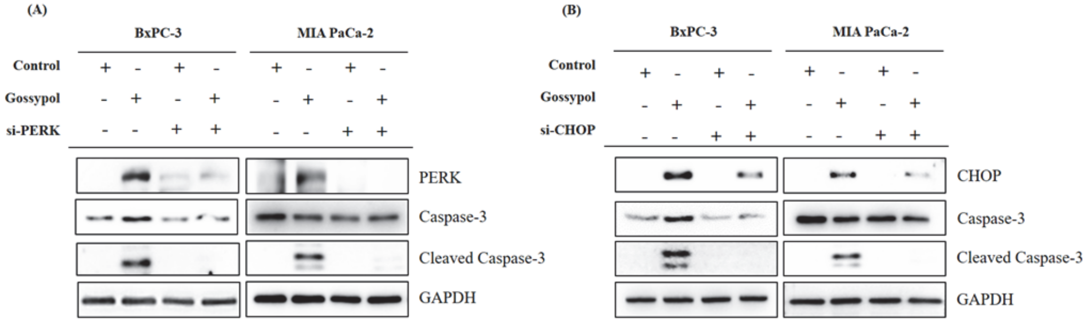

Figure S1. Silencing of PERK and CHOP by siRNA inhibited gossypol treatment from inducing Caspase-3 cleavage in BxPC-3 and MIA PaCa-2 cells. The cells were transfected with siRNA targeting (A) PERK or (B) CHOP, followed by gossypol treatment (10μM) for 24h. The expressional changes of the proteins were analyzed by western blotting.
